# Herd Behavior in Medicine: Examples From COVID-19

**DOI:** 10.7759/cureus.22472

**Published:** 2022-02-21

**Authors:** Haris Iftikhar, Mavia Najam, Khalid Bashir

**Affiliations:** 1 Emergency Medicine, Hamad Medical Corporation, Doha, QAT; 2 Medical Education, Hamad Medical Corporation, Doha, QAT

**Keywords:** telemedicine, fever clinic, drugs, perspective, who, covid-19, herd behavior

## Abstract

Herd behavior is a commonly used term to describe the conduct of different individuals in a group acting without using an individual thought process. The term was first coined by British surgeon Wilfred Trotter in 1914. This editorial will elaborate on how herd behavior has affected the healthcare industry and will include examples from the coronavirus disease 2019 (COVID-19) pandemic. Many interventions were introduced during the initial period of the pandemic, some of them later proved to be either incorrect or only beneficial to a selected group of patients. The nonmedical intervention included personal protective equipment (PPE), the establishment of fever clinics, and telemedicine. Some of these interventions were beneficial and will likely continue after the pandemic. The aim of this editorial is to highlight observed herd behavior in medical practice during pandemics and endorses the need for evaluation of evidence rigorously.

## Editorial

“Herding is a phenomenon by which individuals follow the behavior of others rather than deciding independently on the basis of their own private information” [1]. Herding behavior occurs in animals and humans. Some examples from humans include riots, demonstrations, and escaping during a building fire. The term “Herd behavior” was first coined by British surgeon Wilfred Trotter in 1914. Limited literature is presently available on the role of herding in medical care, such as a neurologist may follow a treatment recommended by a colleague which may not be evidence based [1]. We are writing to discuss how herd behavior has affected the healthcare industry during the COVID-19 pandemic. There is limited literature on the outcomes of herding during the COVID-19 pandemic

Herd behavior can have different perspectives in different settings. During the outset of the COVID-19 pandemic, there appeared to be misunderstandings among healthcare workers and the general public about the pathophysiology and management of the disease. Many treatment options were widely used initially that later showed no benefits or benefits only in a selected group of patients. Several of these treatment options were based on poorly designed studies which were later proved incorrect or currently facing legal action for research misconduct such as chloroquine. Another example is “ivermectin” which was sold as a COVID "miracle" drug that was given to thousands of patients around the world due to studies done by “experts” and other physicians just followed rather than assessing the rigor of the study before prescribing. These and other treatment options were adopted very rapidly all around the world demonstrating the herd behavior in healthcare. Certain antibiotics and anti-inflammatory medications were empirically used generously causing shortages in many countries. The drugs that were initially adopted very rapidly and universally include chloroquine, hydroxychloroquine, azithromycin, oseltamivir, ivermectin, and many others summarized in Table 1 [2].

**Table 1:** Summary of recommendations from WHO living guidelines (8th version) for or against the use of some drugs that were used during the COVID-19 pandemic. WHO definitions for COVID-19 severity: Critical disease is characterized by sepsis, septic shock, acute respiratory distress syndrome, or other conditions due to which the patient needs noninvasive ventilation (NIV) or mechanical ventilation or vasopressor therapy. Severe disease is characterized by room air oxygen saturation <90%, presence of pneumonia on CXR, or signs of severe respiratory distress. Nonsevere or mild disease is characterized by the lack of the above findings for severe or critical disease [2]. IL-6: interleukin 6, CXR: chest X-ray.

Recommendation Type	For/Against	Mild COVID-19	Severe and Critical COVID-19
Strong	For	x	Corticosteroids IL-6 receptor blockers (sarilumab or tocilizumab) baricitinib
Conditional	For	Sotrovimab	x
Conditional	For	Casirivimab and imdevimab	
Strong	Against	Convalescent plasma	x
Strong	Against	Lopinavir-ritonavir, hydroxychloroquine	
Conditional	Against	Corticosteroids	Ruxolitinib and tofacitinib convalescent plasma
Conditional	Against	Remdesivir, ivermectin	

Corticosteroids were used to treat patients during severe acute respiratory syndrome (SARS) and Middle East respiratory syndrome (MERS) epidemics. Initial World Health Organization (WHO) guidelines recommend avoid using corticosteroids for COVID-19 unless there are different indications. The recommendation was based on a meta-analysis that showed corticosteroids were detrimental in patients with SARS. However, the RECOVERY trial on COVID-19 patients who were receiving either invasive mechanical ventilation or oxygen alone found that the use of dexamethasone decreases 28-day mortality. In a study, a prospective meta-analysis of seven clinical trials showed that the use of systemic glucocorticoids reduced the 28-day all-cause mortality [3].

Other treatment options used in COVID-19 include convalescent plasma, awake prone positioning, high-flow oxygen via nasal cannula (HFNC), noninvasive ventilation (NIV) device, intubation and mechanical ventilation, ultraviolet (UV) light, CytoSorb therapy, and mesenchymal stem cells. Convalescent plasma initially widely used all around the world is not recommended except in the clinical trial in severe and critically ill patients [2]. Initial reports did not recommend NIV in COVID-19 as they are aerosol-generating procedures. They can increase the risk of the spread of COVID-19. There were fears that high transpulmonary pressures and large tidal volumes can cause more lung injury. However, retrospective studies showed variable benefits of NIV and HFNC in severe and critically ill COVID-19 patients. HFNC might be a more comfortable and practical mode of support as patients can continue to converse and eat. Both options have their indications, advantages, and disadvantages. In all other treatment options mentioned above, studies are limited and the impact and benefit of these modalities remain unclear.

The concept of fever hospitals is not new in the medical literature. Robert Willan played an essential role in the establishment of the first fever hospital in London in 1802 [4]. With the COVID-19 pandemic, most hospitals made either fever triage areas or clinics to separate fever and respiratory patients from other patients. This rapidly became a global trend demonstrating herd behavior.

Telemedicine is used for a long time. It can be defined as two-way, live communication between the patient and healthcare provider at a distant site using audiovisual equipment. The COVID-19 pandemic led to the widespread implementation of telemedicine and became the standard of continuing care for many outpatient clinics. While telemedicine seems to be an acceptable alternative to most face-to-face outpatient visits, many questions remain unanswered regarding confidentiality, quality of patient care, and healthcare disparities. The global expansion of telemedicine during this pandemic across multiple specialties showed useful aspects of herd behavior in medicine. Although telemedicine utilization declined later in the pandemic after the COVID peaks settled down, it remained markedly higher than the 2018 and 2019 levels. Trends indicate that telemedicine might play a major role in postpandemic healthcare delivery [5].

Conclusion

Herd behavior appears to have a negative and positive impact on medicine and the healthcare industry. COVID-19 pandemic highlights many examples of herd behavior where treatments were disseminated universally which were later found ineffective. Herd behavior has influenced not only the financial markets but also the healthcare industry which may have been driven by pressure from the pharmaceutical industry to increase the sale of certain drugs.

On the other hand, trends such as telemedicine have played a key role in managing patients remotely during pandemic and may continue to support postpandemic healthcare delivery. This editorial strongly recommends identifying herd mentality in medical practice to avoid decisions that might later prove useless in patient's care or even can be harmful to patient's safety.
